# Clinical Spectrum and Treatment Response of Malignant Otitis Externa Patients: A Rural Tertiary Care Centre Experience

**DOI:** 10.7759/cureus.39518

**Published:** 2023-05-26

**Authors:** Shahrukh Ausekar, K C Prasad, Prashanth Babu, Lini Joseph, Induvarsha G

**Affiliations:** 1 Otolaryngology - Head and Neck Surgery, Sri Devaraj Urs Academy of Higher Education and Research, Kolar, IND; 2 Otolaryngology- Head and Neck Surgery, Sri Devaraj Urs Academy of Higher Education and Research, Kolar, IND

**Keywords:** cohen friedman, hrct temporal bone, visual analogue scale, pseudomonas aeruginosa, malignant otitis externa

## Abstract

Introduction: Malignant otitis externa (MOE) is an invasive external ear infection that has a tendency to spread through the temporal bone and can further progress to involve intracranial structures. Though the prevalence of MOE is rare, high morbidity and mortality are often associated. Complications of advanced MOE include cranial nerve involvement, most commonly facial nerve, and intracranial infections such as abscesses and meningitis.

Materials and methods: In this retrospective case series of nine patients diagnosed with MOE, demographic data, clinical presentations, laboratory data, and radiological findings were reviewed. All patients were followed up for a minimum period of three months after discharge. Outcomes were measured in terms of reduction in obnoxious ear pain (Visual Analogue Scale), ear discharge, tinnitus, need for re-hospitalization, recurrence of disease, and overall survival.

Results: In our case series of nine patients (seven males and two females), six underwent surgery, and three patients were managed with a medical line of treatment. All patients had a significant reduction in otorrhea, otalgia, random venous blood sugars, and improvement of facial palsy implicating good response to treatment.

Conclusion: Prompt diagnosis of MOE warrants clinical expertise and aids in preventing complications. A prolonged course of intravenous anti-microbial agents is the mainstay of treatment, but timely surgical interventions in treatment-resistant cases can prevent complications.

## Introduction

Malignant otitis externa (MOE) is a misnomer since it is not a neoplastic process [1]. It implies an aggressive inflammation of the external auditory canal in elderly diabetic, and immunocompromised individuals with *Pseudomonas aeruginosa* as the main culprit followed by methicillin-resistant *Staphylococcus aureus* and other fungal organisms [2,3]. The inflammatory process is so fierce that it may involve the skull base leading to Skull Base Osteomyelitis [3]. The sequelae of this catastrophic condition can result in facial nerve palsy in adjunction to other lower cranial nerve palsies leading to deterioration in the quality of life [4-7].

Radiological investigations in the form of computed tomography (CT) with intravenous contrast are of paramount importance, aiding in diagnosis and understanding the pathological involvement and extent of the disease process [8,9]. In the era of modern and wide-spectrum antibiotics, the role of surgery has receded and long-term antibiotics have become the first line of treatment [10-12]. Surgical procedures range from local debridement to radical and extended mastoidectomy with prior biopsy of granulation tissue to rule out malignancy [11,12]. Poor diabetic control, prolonged hospital stay, inevitable treatment expenses and reduced patient compliance to treatment are the main limiting factors for treatment and disease cure [13-15].

However, decision-making with regard to management is still debatable as there is no definitive treatment protocol. We aimed to describe the clinical presentation, diagnostic methods, treatment response, and quality of life in patients with MOE who presented to our hospital.

## Materials and methods

This retrospective observational study was conducted in Sri Devaraj Urs Academy of Higher Education and Research, Kolar, between December 2021 and April 2022. The study was approved by the Institutional Ethics Committee (approval number IEC No DMC/KLR/IEC/ 397/2022-23). All patients diagnosed with MOE fulfilling Cohen-Friedman criteria were included in the study.

Cohen-Friedman criteria: Obligatory: Pain, edema, exudate, granulation tissues, micro abscess, positive bone scans, failure of local treatment for more than a week, and possibly pseudomonas in culture. Occasional: Diabetes, cranial nerve involvement, positive radiographic scans, debilitating conditions, and old age.

Patients with intracranial complications, malignancy of temporal bone, bleeding diathesis, and patients with acute ear trauma were excluded from the study. Written informed consent was obtained from the study participants to use their clinical data. The presenting symptoms, past history, clinical examination findings including oto-microscopy findings, radiological details, and laboratory parameters of these patients were reviewed. Patients received both medical as well as surgical modalities of treatment.

All medical and surgical data of patients were reviewed and documented. All methods for glycemic controls were reviewed and documented. Outcomes were measured in terms of reduction in excruciating ear pain using Visual Analogue Scale (VAS), ear discharge, ringing sensation, need for re-hospitalization, recurrence of disease, and survival. Pain scale and duration of hospital stay were reviewed. Patients were followed up for a minimum period of three months and oto-microscopic findings along with patient complaints were documented and addressed.

Statistical analysis

Data was entered into a Microsoft (MS) Excel data sheet and analyzed using SPSS software, version 22 (IBM Corp., Armonk, NY). ANOVA test was done to signify a reduction in pain as compared to the time of presentation. In accordance with the ANOVA test, the k and p values were calculated.

Graphical Representation of Data

MS Excel and MS Word were used to obtain various graphs. A p-value of <0.05% was considered statistically significant.

## Results

The total number of patients included in our study was nine. Amongst these, seven were male and two were female. The mean age of patients was 58 years. The majority population under study was diabetic (88.8%), followed by hypertensive (44.4%), ischemic heart disease (22.2%), and chronic kidney disease (1.1%). One patient on routine investigations was found to be HIV positive (Table 1). Ear pain was the common complaint for all the patients which was a throbbing type, aggravated at night, not relieved by oral analgesics that obliged frequent administration of intra venous analgesics (tramadol thrice per day) and mean VAS score at the time of presentation was eight. The highest score was nine and the lowest was seven as shown in Table 1.

**Table 1:** Demographic data and pre-operative findings VAS: Visual Analogue Scale

Patients	N= 9
Age	58 years
Male	07
Female	02
Diabetes	08
Hypertensive	04
Ischemic Heart Disease	02
Chronic Kidney Disease	01
HIV	01
Otalgia	09 (Mean VAS= 8 )
Otorrhea	09
Reduced hearing	07
Tinnitus	06
External canal oedema	09
Tragal Tenderness	09
Discharge	09
Granulation tissue	09
Aural Polyp	04
Facial Nerve Palsy	02 (House Brackman Grade 4)
Pre Auricular swelling	01

Otorrhea was seen in nine patients, which was thick, mucopurulent, moderate in amount, foul smelling, and non-blood-tinged. Pus from the external auditory canal was sent for culture and sensitivity on the day of admission. Later, aural toileting was performed once in two days under an oto-microscope.

Decreased hearing in the diseased ear was reported in seven patients. Pure tone audiometry revealed three patients had moderately severe sensorineural hearing loss (56-70 dB HL) while four patients had mild hearing loss (26-40 dB HL). Tinnitus in the affected ear was seen in six patients, which was subjective in nature, more during night time, aggravated with episodes of ear discharge, and had no relieving factors.

All patients who presented to our outpatient department with the above-mentioned complaints underwent a detailed ENT examination. Tragal tenderness was noted in all patients. Upon oto-microscopy, canal edema was present in all patients and granulation tissues were present over the bony-cartilaginous junction in all patients.

Aural polyps were seen in four patients. Polyps were protruding through the external ear and completely occluded the external auditory canal. Pre-auricular swelling was seen in one patient due to parotid involvement leading to abscess formation along with facial nerve palsy. Facial nerve weakness (House Brackman Grade 4) was seen in two patients at the time of presentation. Both these patients were diabetic.

Patients were diagnosed with MOE by Cohen-Friedman criteria and were admitted. Pus from the ear discharge was sent for Gram stain and culture sensitivity. All patients underwent relevant blood tests, audiological tests (pure tone audiometry), and radiological examination (high-resolution computed tomography {HRCT} of temporal bone). Pre-intervention glycosylated hemoglobin was 9.65% indicating poor diabetic control.

Patients were empirically started on a combination of intravenous ciprofloxacin 500 mg with piperacillin + tazobactam 4.5 gm thrice daily. Later, antibiotics were administered as per culture and sensitivity results. Culture reports revealed *Pseudomonas aeruginosa* in the majority of patients (77.77%) followed by *Candida* and *Enterococcus*.

One patient had chronic kidney disease, antibiotic regimen was initiated on the advice of a nephrologist since the patient was undergoing dialysis. The patient was treated with intravenous ceftazidime 1 gram twice daily along with serial monitoring of renal parameters.

A history of Ischemic heart disease was present in one patient. Two-dimension echocardiogram showed no new changes. One patient had pre-auricular swelling, ultrasonography showed 20 cc of pus that was surgically drained. Another HIV-positive patient was initiated on anti-retroviral drugs in addition to anti-pseudomonas drugs.

Along with our aggressive approach with anti-microbial agents, all patients received injectable analgesic (tramadol thrice daily) and injectable insulin (short-acting human actrapid + long-acting glargine). To assess the alleviation of pain, the frequency of administrating intravenous analgesics was noted.

Otorrhea subsided with the instillation of antibiotic ear drops (acetic acid drops, ciprofloxacin ear drops) and intravenous antibiotics. Aural toileting was performed under an oto-microscope once in two to three days.

HRCT of temporal bone revealed soft tissue densities in the middle ear cavity, epitympanic recess, Prussack’s space, attic, aditus, antrum, mesotympanum, hypotympanum, external auditory canal, and Eustachian tube. Erosion of scutum, tegmen, with ossicles was noted. The mastoid was sclerosed.

Based on the patient profile, the extent of existing pathology, and inadequate response to the medical line of treatment, six patients underwent surgical modality of treatment with canal wall down mastoidectomy in addition to incision and drainage of a pre-auricular abscess in one patient. The remaining three patients were managed by medical line as two patients responded well and one patient did not give consent for surgery as shown in Table 2.

**Table 2:** Modalities of treatment of patients under study

Management	Number of patients
Medical management alone	03
Surgical+ Medical management	06
Total	09

Prior to surgery, physician opinion was sought and relevant investigations were performed as a part of the pre-anesthetic check-up, and patients were taken up for surgery. A detailed written informed consent explaining the nature of the procedure was obtained from all the participants.

**Figure 1:**
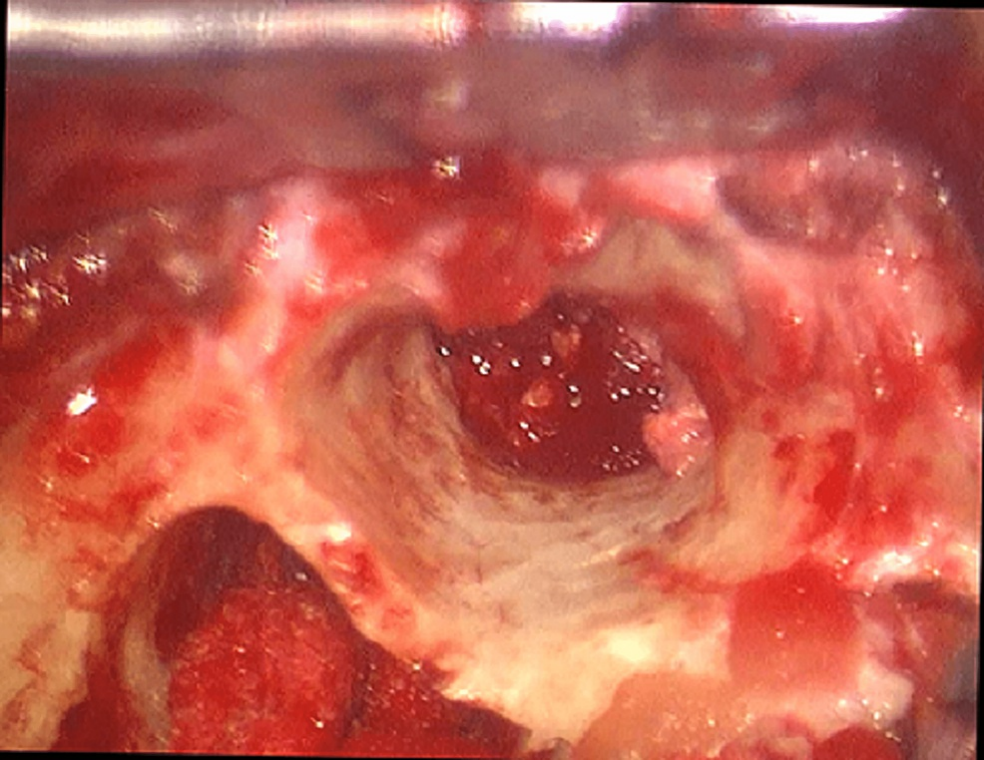
Granulation tissues in the middle ear

Canal wall down mastoidectomy was opted to debride the granulation tissues and to know the extent of polypectomy in six patients. Intra-operative findings included the presence of granulation tissue in the external canal, eustachian tube, facial canal, and middle ear in six patients (83.3%) as shown in Figure 1.

Patients were monitored in step-down intensive care for a day and later were shifted to the parent ward. The medical line of treatment was continued in the post-operative period. Post-operative day seven mastoid dressing was removed, and on day 10, post-aural sutures were removed. Later on post-operative day 15, the canal pack was removed and the neo cavity was examined.

There was not much difference noted between the medical and surgical lines of management in terms of the resolution of otalgia and otorrhea. Otalgia was assessed by the need to administer intravenous analgesics while otorrhea was evaluated by dry canal. We managed to reduce otalgia by 12 days and otorrhea by 10 days after the initiation of treatment. 

Patients were discharged after a reduction in the severity of symptoms. None of the patients had a recurrence. The average duration of hospital stay was 29.66 days. Patients were called monthly for three consecutive months following discharge.

On every follow-up, patients were subjected to oto microscopy and findings were registered. Since the majority of patients underwent Canal wall down mastoidectomy, canal-related problems (ear discharge, pus from post-auricular wound, granulation tissues) were addressed, and in the post-operative period, patients were advised to continue antibiotic ear drops.

In our study, patient compliance was observed till the last follow-up visit. The mean pain score at the time of presentation was eight. With the initiation of treatment, the score reduced significantly. At the subsequent follow-up visit of three months, VAS was 2 as shown in Table 3.

**Table 3:** Comparison between average VAS score at the time of admission, discharge and following subsequent visits VAS: Visual Analogue Scale

Mean VAS Score Admission	Mean VAS Score Discharge	Mean VAS Score 1 month	Mean VAS Score 2 month	Mean VAS Score 3 month
8	3	3.3	2.6	2

ANOVA test was applied to know the p-value (<0.05%). F-value was 1525.25 signifying a p-value less than 0.0001% which was statistically significant. Till the last follow-up visit, all patients were free of pain. Otorrhea was not observed in any patients on follow-up visits. Tinnitus intensity and number of episodes were lowered, but persisted in four patients and were managed symptomatically.

Patients with decreased hearing had fair responses owing to the sensorineural component and they were advised for a hearing aid. Facial nerve weakness showed a slight improvement on the House Brackman scale of 4 to 2 with physiotherapy exercises.

In the last follow-up visit, we were able to achieve a healthy cavity with adequate epithelization. Radiological exams were not performed post-operatively since all patients had a good response and had no complaints or features suggesting recurrence on follow-up otological examination. There was no re-appearance of symptoms, recurrence of the disease, or need for re-hospitalization. There was 100% survival noted.

## Discussion

Toulmouche reported the first case of malignant otitis externa. Later, Chandler in 1968 coined the term ‘Malignant Otitis Externa’ due to its aggressive nature, delayed diagnosis, and high incidence of morbidity and mortality [1].

Symptoms of malignant otitis externa include excruciating otalgia, otorrhea, tinnitus, facial nerve palsy, and in advanced stages catastrophic outcomes if a contralateral skull base is involved [2]. The hallmark of this condition is the presence of granulation tissues over the bony cartilaginous junction. Due to the overlapping features with other diseases like otitis externa, carcinoma of the ear canal, and chronic otitis externa, diagnosis is challenging. Microbiological cultures and radiological investigations are performed in adjunction to confirm the diagnosis [2].

The most accepted criteria for diagnosing malignant otitis externa was proposed by Cohen and Friedman [1]. The pathogenesis begins as a soft tissue infection of the external auditory canal which spreads to the temporal bone and skull base, leading to granulation tissue formation further involving petrous soft tissues. The facial nerve is often involved. Sigmoid sinus involvement causes retrograde thrombophlebitis [3,7]. Intracranial extension results in the involvement of opposite skull bases [1,7].

Though it is a known entity since antiquity, there are limitations in a standard treatment protocol making it a debatable issue [1]. We undertook this study with the aim of documenting varied presentations, treatment challenges, the need for a multidisciplinary approach, and response evaluation to our treatment. 

In our study, the majority of the patients were male, in contrast to a study done by Yigider et al. that had a maximum female population [4]. Earlier literature had shown a preponderance of the diabetic male population, but the recent literature shows a shifting trend. The average age of the population under our study was 58 years in contrast to other studies that reported between 67-83 years [2-4]. Eweiss et al., in their study, concluded that aging was no longer a factor for its incidence [3]. Excruciating ear pain was the most troublesome symptom, similar to other studies. Others included otorrhea, decreased hearing, tinnitus, and facial nerve palsy. In our study, facial nerve palsy (House Brackman Grade 4) was seen in 22.2% of patients in contrast to 26.9% reported by Byun et al. [9] and 32.37% of patients reported by Dabiri et al. [14].

We encountered varied comorbid pathologies in our study, type 2 diabetes being the most common condition followed by hypertension, ischemic heart disease, chronic kidney disease, and HIV. In our study seven patients were diabetic. Studies done by Trevino Gonzalez et al. [5], Peled et al. (2020) [6], and Yang et al. [15] quoted a positive relationship between diabetes and malignant otitis externa. Poor diabetic control implicates prolonged hospital stay as noted by Peled et al. (2022) [15]. Non-compliance to treatment, dietary modification, stress, and insulin resistance may have contributed to uncontrolled sugars in these patients.

Microbiological cultures concluded *Pseudomonas* as the most common agent (77.7%). Chen et al. showed a decreased incidence of pseudomonas aeruginosa (26.9%) [7]. However, there is a rise in trend of fungal and other organisms that have poor prognosis [2]. In our study, a single case showed Candida and Enterococcus responded well to antimicrobials and antifungal agents were not needed. 

Laboratory investigations are often sought that include C-reactive protein, erythrocyte sedimentation rate, and differential leucocyte count. But there is limited evidence of these being sensitive in diagnosing malignant otitis externa [7,16]. In our study, we did not perform these tests owing to financial constraints.

Radiological investigations like Tc99m and Ga67 help diagnose and monitor disease progression however have their limitations in the form of high cost, radiation exposure, and unavailability [5]. Computed tomography (CT) and magnetic resonance imaging with contrast provide better anatomical resolutions but are not sensitive and specific as standard modalities [5]. 2-Deoxy-2-fluoro-D glucose integrated with CT yields a promising modality to assess treatment response, hence future trials are warranted [5]. A meta-analysis by Moss et al. showed the sensitivity of Technicium 99m was 85% while that of Gallium 67 was 71.2% [8]. A systematic review by Termat et al. showed that 2-deoxy-2-fluoro-D glucose Positron Emission Tomography had a sensitivity of 96% and specificity of 91% [5,8]. In our study, high-resolution CT was performed to assess the extent of the disease process. Similarly, a study conducted by Long et al. concluded that computed tomography yields excellent results in indicating bony involvement but has poor outcomes in terms of disease monitoring [7]. Honurappa et al. implicated HRCT temporal bone in their study on 51 patients for diagnosis and following treatment to evaluate the response [17].

Long-term treatment with anti-pseudomonal antibiotics plays a pivotal role in combating the disease. All our patients responded well to our combination therapy (ciprofloxacin 500 mg with piperacillin + tazobactam 4.5 gm). Other potent agents quoted in the literature include ciprofloxacin, ceftazidime, and meropenem [7,9].

Although medical line prevails as the standard treatment, there is an increase in trends toward surgical modality [6]. Peled et al. viewed surgical intervention in 90% of the cases under his study and had favorable outcomes. In our study, six cases underwent surgical modality of treatment while the remaining were managed with a medical line of management. There was no significant difference in response to both modalities.

**Figure 2:**
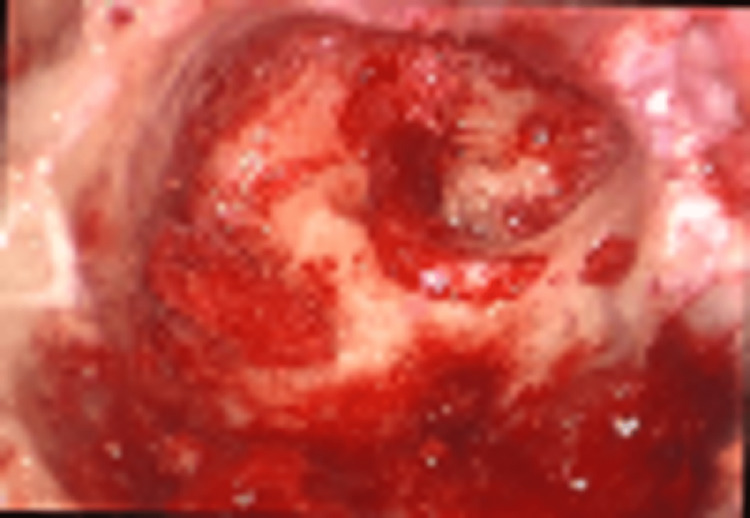
Intra-operative facial nerve involvement

The intra-operative findings were not only suggestive of the disease but also were imperative of extensive involvement similar to a study done by Peled et al. [6]. In our study, one patient had a parotid abscess and underwent incision and drainage. The facial nerve was involved in two cases; hence decompression was performed as shown in Figure 2.

Post-operative outcomes were favorable and none of the patients had any complications. The average in-patient stay was 4.1 weeks in contrast to Mehdi et al. study that reported six weeks [10]. On follow-up visits, our patients had alleviation of troublesome symptoms that were indicated by a decrease in the frequency of analgesics, reduced VAS score, and dry ear.

Our holistic approach not only rendered patients disease free (100% success) but also led them to have a better quality of life. This was indicated by the reduction in baseline symptoms, absence of recurrences, no readmissions, and no deaths. A similar study was done by Arsovic et al. concluded 7% mortality and 23% recurrence in their 10 years of follow-up [11]. In a case series of 14 patients by Mariana et al., the relapse rate was 21% [16]. Similarly, Honnurappa et al. reported 90% success and concluded that diabetic control is crucial [17].

Though the disease is known for ages, there is a deficiency in knowledge regarding diagnosis and treatment protocol globally [5]. Observed incidence is merely the tip of the iceberg [5]. There is no standard accepted protocol for managing the disease [12]. There is wide variation in outcomes measured by conducting studies across the globe.

The limitations of our study were the relatively small sample size, short follow-up duration, and retrospective nature of the study. Hence, to emphasize a protocol, large prospective cohort studies with longer follow-up periods should be undertaken.

## Conclusions

Malignant otitis externa often mimics other ear pathologies encountered in clinical practice. Hence, its diagnosis can be a challenge. Due to the wide spectrum of clinical presentation and relatively unpredictable course of the disease, decision making regarding treatment requires expertise and experience in managing this dreadful condition. A prolonged course of intravenous antibiotics is the mainstay of treatment. Disease aggressiveness and poor response to antibiotics warrant surgical intervention. In such cases, medical and surgical intervention has to be done synchronously to achieve a good response. Even in a rural tertiary care centre with limited resources and economic constraints, successful treatment and good survival outcome is possible through early diagnosis and prompt intervention.
